# Supplementary material for: The Systems Biology Research Tool: evolvable open-source software
Source: BMC Syst Biol. 2008 Jun 29;2:55. doi: 10.1186/1752-0509-2-55 (PMC2446383; doi:10.1186/1752-0509-2-55)
Supplement: Additional file 1 — SBRT Archive. An archive of the current version of the Systems Biology Research Tool. [file 1752-0509-2-55-S1.zip › sbrt-1.4.0/doc/developers_guide/api/sbrt/kernel/fba/proc/CvRxnDeletionProcess.html]

CvRxnDeletionProcess


|  |  |  |
| --- | --- | --- |
| **Overview** \| **Package** \| **Class** \| **Use** \| **Tree** \| **Deprecated** \| **Index** \| **Help** | |  |
| **PREV CLASS**   **NEXT CLASS** | **FRAMES**    **NO FRAMES**     **All Classes** |  |
| SUMMARY: NESTED \| FIELD \| CONSTR \| METHOD | DETAIL: FIELD \| CONSTR \| METHOD |  |


---


## sbrt.kernel.fba.proc Class CvRxnDeletionProcess

```
java.lang.Object
  sbrt.kernel.fba.proc.FbaProcess<FbaOptComputer.Results>
      sbrt.kernel.fba.proc.CvRxnDeletionProcess
```

**All Implemented Interfaces:**: KernelProcess<FbaOptComputer.Results>, MonitoredProcess<FbaOptComputer.Results>

---

``` public final class CvRxnDeletionProcess extends FbaProcess<FbaOptComputer.Results> implements MonitoredProcess<FbaOptComputer.Results> ```

This class is used to perform constraint-variation reaction deletion
experiments.

**Author:**
:   This class was written and documented by Jeremiah Wright while in the
    Wagner lab.

---

**Constructor Summary**

`CvRxnDeletionProcess(RxnDeletionComputer computer)`
  
          Constructs a new constraint-variation reaction deletion experiment.


| **Method Summary** | |
| --- | --- |
| `static void` | `checkForConflict(FluxConstraints fc, java.util.Set<java.lang.String> deletionNames)`             Ensures that the provided flux constraints and set of reaction names do not conflict. |
| `Fluxome` | `getFluxome()`             Returns the fluxome used by the reaction deletion computer. |
| `MathExpr<java.lang.String>` | `getObjectiveFunction()`             Returns the current objective function. |
| `OptimizationSense` | `getOptimizationSense()`             Returns the current optimization sense. |
| `ProgressMonitor` | `getProgress()`             Returns a progress monitor for this experiment. |
| `double` | `getZeroCutoff()`             Returns the current zero cutoff used by the reaction deletion computer. |
| `void` | `run(ResultsManager<FbaOptComputer.Results> resultsManager)`             Runs this process and adds the generated results to the provided results manager via its `ResultsManager.addData(Object)` method. |
| `void` | `setConstraintSource(java.lang.Iterable<FluxConstraints> constraintSource)`             Sets the constraint variation input source. |
| `void` | `setObjectiveFunction(MathExpr<java.lang.String> objective)`             Sets the objective function to the provided mathematical expression. |
| `void` | `setOptimizationSense(OptimizationSense sense)`             Sets the optimization sense to the provided value. |
| `void` | `setRxnDeletionSource(java.lang.Iterable<java.util.Set<java.lang.String>> deletionSource)`             Sets the reaction deletion input source. |
| `void` | `setZeroCutoff(double zeroCutoff)`             Sets the zero cutoff to the provided value. |

**Methods inherited from class sbrt.kernel.fba.proc.FbaProcess**

`toString`

**Methods inherited from class java.lang.Object**

`clone, equals, finalize, getClass, hashCode, notify, notifyAll, wait, wait, wait`

**Constructor Detail**

### CvRxnDeletionProcess

```
public CvRxnDeletionProcess(RxnDeletionComputer computer)
```

:   Constructs a new constraint-variation reaction deletion experiment.

    **Parameters:**: `computer` - the computer with which all results will be computed.


**Method Detail**

### getFluxome

```
public Fluxome getFluxome()
```

:   Returns the fluxome used by the reaction deletion computer.

    :   **Specified by:**: `getFluxome` in class `FbaProcess<FbaOptComputer.Results>`
    :   **Returns:**: the fluxome used by the reaction deletion computer.

---


### getZeroCutoff

```
public double getZeroCutoff()
```

:   Returns the current zero cutoff used by the reaction deletion computer.

    :   **Returns:**: the current zero cutoff used by the reaction deletion computer.

---


### getObjectiveFunction

```
public MathExpr<java.lang.String> getObjectiveFunction()
```

:   Returns the current objective function.

    :   **Returns:**: the current objective function.

---


### getOptimizationSense

```
public OptimizationSense getOptimizationSense()
```

:   Returns the current optimization sense.

    :   **Returns:**: the current optimization sense.

---


### setObjectiveFunction

```
public void setObjectiveFunction(MathExpr<java.lang.String> objective)
```

:   Sets the objective function to the provided mathematical expression.

    :   **Parameters:**: `objective` - a mathematical expression of reaction names.

---


### setOptimizationSense

```
public void setOptimizationSense(OptimizationSense sense)
```

:   Sets the optimization sense to the provided value.

    :   **Parameters:**: `sense` - an optimization sense.

---


### setRxnDeletionSource

```
public void setRxnDeletionSource(java.lang.Iterable<java.util.Set<java.lang.String>> deletionSource)
```

:   Sets the reaction deletion input source.

    :   **Parameters:**: `deletionSource` - a source of reaction names sets whose effect upon deletion is to be computed.

---


### setConstraintSource

```
public void setConstraintSource(java.lang.Iterable<FluxConstraints> constraintSource)
```

:   Sets the constraint variation input source.

    :   **Parameters:**: `constraintSource` - a source of constraint sets whose effect upon application is to be computed.

---


### setZeroCutoff

```
public void setZeroCutoff(double zeroCutoff)
```

:   Sets the zero cutoff to the provided value. The zero cutoff is the
    value used to decide which computed values are close enough to zero
    to be considered equal to zero. This value is an upper bound and
    should itself be close to zero, like 1E-6 for example.

    :   **Parameters:**: `zeroCutoff` - the zero cutoff.

---


### getProgress

```
public ProgressMonitor getProgress()
```

:   Returns a progress monitor for this experiment.

    :   **Specified by:**: `getProgress` in interface `MonitoredProcess<FbaOptComputer.Results>`
    :   **Returns:**: a progress monitor for this experiment.

---


### run

```
public void run(ResultsManager<FbaOptComputer.Results> resultsManager)
```

:   Runs this process and adds the generated results to the provided results
    manager via its `ResultsManager.addData(Object)` method.

    First, a set of flux constraints is retrieved from the constraint source
    and applied. Then a set of reaction names in the deletion source is
    retrieved. The results are computed and added to the results manager.
    Each set of reaction names is iterated through in the same fashion. When
    the end of the deletion source is reached, the flux constraints are
    restored to their original value and `resultsManager.breakPoint()`
    is called. At this point, the next set of flux constraints is retrieved
    from the constraint source. The entire process is repeated until the end
    of the constraint source is reached.

    :   **Specified by:**: `run` in interface `KernelProcess<FbaOptComputer.Results>`
    :   **Parameters:**: `resultsManager` - the results manager to which the results will be added.

---


### checkForConflict

```
public static void checkForConflict(FluxConstraints fc,
                                    java.util.Set<java.lang.String> deletionNames)
```

:   Ensures that the provided flux constraints and set of reaction names do
    not conflict.

    :   **Parameters:**: `fc` - a set of flux constraints.: `deletionNames` - a set of reaction names. **Throws:**: `ConflictingInputException` - if reaction names occur in both the provided flux constraints and set of reaction names. A list of common reaction names can be retrieved with `ConflictingInputException#getConflicts()`.


---


|  |  |  |
| --- | --- | --- |
| **Overview** \| **Package** \| **Class** \| **Use** \| **Tree** \| **Deprecated** \| **Index** \| **Help** | |  |
| **PREV CLASS**   **NEXT CLASS** | **FRAMES**    **NO FRAMES**     **All Classes** |  |
| SUMMARY: NESTED \| FIELD \| CONSTR \| METHOD | DETAIL: FIELD \| CONSTR \| METHOD |  |


---
